# Postoperative atrial fibrillation in patients with left atrial myxoma

**DOI:** 10.5830/CVJA-2014-069

**Published:** 2015

**Authors:** Muslum Sahin, Cihan Dundar, Gokhan Alici, Serdar Demir, Mehmet Emin Kalkan, Birol Ozkan, Kursat Tigen, Beste Ozben

**Affiliations:** Department of Cardiology, Kartal Kosuyolu Heart Education and Research Hospital, Istanbul, Turkey; Department of Cardiology, Kartal Kosuyolu Heart Education and Research Hospital, Istanbul, Turkey; Department of Cardiology, Kartal Kosuyolu Heart Education and Research Hospital, Istanbul, Turkey; Department of Cardiology, Kartal Kosuyolu Heart Education and Research Hospital, Istanbul, Turkey; Department of Cardiology, Kartal Kosuyolu Heart Education and Research Hospital, Istanbul, Turkey; Department of Cardiology, Kartal Kosuyolu Heart Education and Research Hospital, Istanbul, Turkey; Department of Cardiology, Marmara University School of Medicine, Istanbul, Turkey; Department of Cardiology, Marmara University School of Medicine, Istanbul, Turkey

**Keywords:** atrial fibrillation, left atrium, myxoma, postoperative, P-wave dispersion

## Abstract

**Introduction:**

The aim of this study was to determine the factors associated with postoperative atrial fibrillation (AF) in patients with left atrial (LA) myxoma.

**Methods:**

Thirty-six consecutive patients with LA myxoma (10 men, mean age: 49.3 ± 15.7 years), who were operated on between March 2010 and July 2012, were included in this retrospective study. Pre-operative electrocardiograms and echocardiographic examinations of each patient were reviewed.

**Results:**

Postoperative AF developed in 10 patients, whereas there was no evidence of paroxysmal AF after resection of the LA myxoma in the remaining 26 patients. The patients who developed AF postoperatively were significantly older than those who did not develop AF (median: 61.5 vs 46 years; *p* = 0.009). Among the electrocardiographic parameters, only P-wave dispersion differed significantly between postoperative AF and non-AF patients (median: 57.6 vs 39.8 ms, *p* = 0.004). Logistic regression analysis revealed P-wave dispersion (OR: 1.11, 95% CI: 1.003–1.224, *p* = 0.043) and age (OR: 1.13, 95% CI: 1.001–1.278, *p* = 0.048) as independent predictors of postoperative AF in our cohort of patients.

**Conclusions:**

P-wave dispersion is a simple and useful parameter for the prediction of postoperative AF in patients with LA myxoma.

## Abstract

Paroxysmal atrial fibrillation (AF) is the most common arrhythmia following cardiac surgery such as coronary artery bypass grafting (CABG), and often occurs between the second and fourth postoperative days.^1^,^2^ The reported incidence of paroxysmal AF after CABG surgery varies widely, from five to 40%, which is lower than in cases of valvular cardiac surgery.^3^,^4^ Although this arrhythmia is usually benign and self-limiting, it may also be associated with increased risk of embolic events, haemodynamic instability, haemorrhagic complications, prolonged hospital stay and higher rates of re-admissions, increasing the healthcare costs.^5^-^7^

Several risk factors have been proposed for paroxysmal AF after CABG or valvular cardiac surgery, such as advanced age, genetic predisposition, chronic obstructive pulmonary disease, heart failure or increased peri-operative ischaemia.^8^-^10^ In addition, certain echocardiographic parameters such as left atrial (LA) diameter or left ventricular (LV) function, and electrocardiographic parameters including P-wave duration and P-wave dispersion (Pd) have been shown to be associated with postoperative AF.^11^-^13^

Although postoperative AF and its predictors after CABG and valvular surgery have been well researched, no study has been performed to explore the incidence or predictors of postoperative AF in patients with LA myxoma. The aim of this study was to identify the prevalence and predictors of postoperative AF in a pure cohort of patients with LA myxoma.

## Methods

This study complies with the principles outlined in the Declaration of Helsinki. The study was approved by the local ethics committee and all participants gave written informed consent to participate in the study.

The electrocardiograms and echocardiographic recordings of the 44 consecutive patients with LA myxoma who underwent its excision in our centre between March 2000 and July 2012 were evaluated retrospectively. Previous history of AF or atrial flutter, use of anti-arrhythmic drugs other than beta-blockers, concomitant valvular disease other than mild mitral regurgitation, symptomatic heart failure, renal disease, thyroid disorders, chronic obstructive pulmonary disease, and presence of an implanted pacemaker were exclusion criteria. Patients who had undergone any surgery other than excision of a LA myxoma, including CABG, had sustained ventricular tachyarrhythmia or cardiogenic shock or died in the operating room were also excluded.

All medical records including standard pre-operative 12-lead electrocardiograms (ECG), transthoracic echocardiography, laboratory tests and blood pressure measurements were carefully checked and documented. All patients were in sinus rhythm before surgical excision of the tumour and their cardiac rhythms were followed continuously during their stay in the intensive care unit for at least for 48 hours by direct rhythm monitoring.

After discharge from the intensive care unit, the patients were followed up with daily ECGs and rhythm evaluation after complaints of palpitations, to diagnose any episodes of paroxysmal AF. All patients were re-evaluated three months after surgery and the ECG and echocardiographic examinations performed at that visit were also recorded.

After the exclusion of patients with any missing information, the remaining 36 out of 44 patients with LA myxoma who were surgically treated in our institution were included in the study. Postoperative AF was defined as any episode of atrial tachyarrhythmia, including AF or atrial flutter that lasted more than 30 seconds, diagnosed with a rhythm monitor/telemetry and/or ECG, and/or initiation of treatment for atrial fibrillation such as amiodarone or cardioversion during hospitalisation.^14^,^15^

Surgery was performed via a median sternotomy under cardiopulmonary bypass with cardioplegic arrest. The LA myxoma was excised through a left atriotomy with trans-septal approach or via a biatrial approach in suitable cases. After removing the mass, the resulting atrial septal defect was repaired by direct suture or insertion of a Dacron patch.

## Evaluation of pre- and postoperative ECGs

All patients had standard pre-operative (one day before surgery) and postoperative (one week after surgery) 12-lead ECGs, which were recorded at a paper speed of 25 mm/s, a sensitivity of 1 mV/cm and filter settings of 0.05–40 Hz. The ECGs were scanned and magnified five times.

P-wave duration and dispersion were measured as previously described.^16^ Briefly, P-wave duration was measured in three consecutive complexes of each lead, from the junction between the iso-electric line and the beginning of the P-wave deflection to the junction between the end of the P wave and isoelectric line, by a single observer who was blinded to the patients. To improve accuracy, measurements were made using calipers and a magnifying lens. P-wave dispersion was defined as the time measured from the onset to the offset of the P wave.

The P_max_ and the P_min_ were measured in all 12-lead surface ECGs. The Pd was defined as the difference between the P_max_ and the P_min_. Intra-observer variability was found to be 4.5% for P_max_ and 4.1% for Pd. A Pd > 40 ms was defined as increased Pd.^17^ The P–R interval, QRS duration, QT and rate-corrected QT interval were measured similar to previous studies.^18^,^19^

## Evaluation of echocardiography

All patients underwent transthoracic echocardiography, performed according to American Society of Echocardiography recommendations before surgery and three months after surgery.^20^ LA diameter and LV dimensions of the patients obtained by M-mode echocardiography in the parasternal long-axis view were recorded. Mitral regurgitation (MR) was graded by standard Doppler criteria.

**Figure 1.**
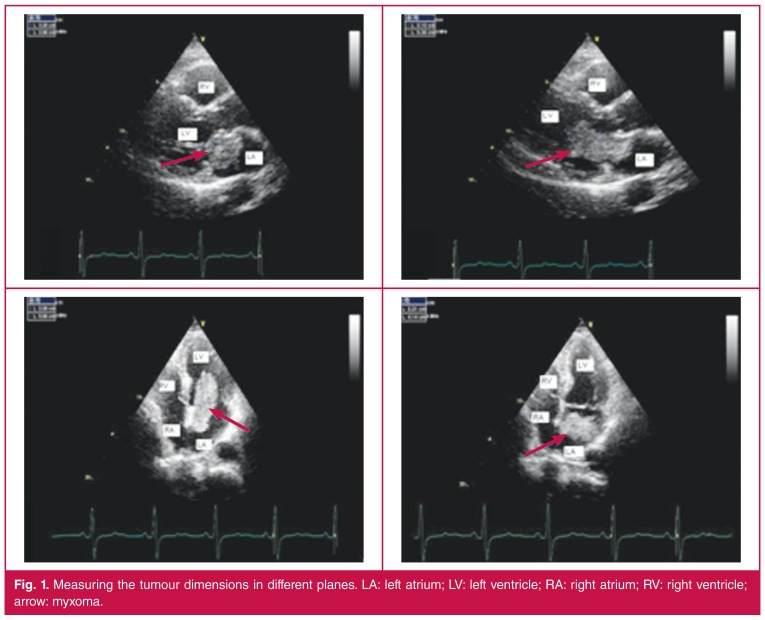
Measuring the tumour dimensions in different planes. LA: left atrium; LV: left ventricle; RA: right atrium; RV: right ventricle; arrow: myxoma.

Tumour dimensions were measured in three different planes. The maximum diameter in any of the planes was taken as a reference of the size of the tumour in that plane (Fig. 1). By calculating the average radius of the tumour in three different planes, the approximate echocardiographic volume of the tumour was calculated using the formula 4/3πr^3^.^21^

## Statistical analysis

Statistical analysis was performed using a statistical software program (SPSS for Windows, version 15.0; SPSS Inc, Chicago, Illinois, USA). Continuous variables were expressed as medians (min–max), controlled for normal distribution by the Kolmogorov–Smirnov test and compared using non-parametric tests (Mann–Whitney *U*-test) because of abnormal distribution. Categorical data between two or more groups were compared with the Pearson χ^2^ test. Pre- and postoperative ECG data were compared with the Wilcoxon test. A logistic regression analysis was used to determine significant predictors of postoperative AF in patients with LA myxoma. A *p*-value < 0.05 was considered statistically significant.

## Results

The study included 36 consecutive patients with LA myxoma (10 men, mean age: 49.3 ± 15.7 years). The most commonly reported symptom was dyspnoea, which was observed in 13 patients. Eight patients presented with palpitations, three with angina, five complained of syncope and seven had a transient ischaemic attack or cerebrovascular event. Seven patients were asymptomatic.

The LA myxoma was excised through a left atriotomy in 19 patients, whereas the trans-septal and biatrial approach were used in the remaining nine and eight patients, respectively. After removing the mass, the resulting atrial septal defect was repaired by direct suture in 34 patients and by insertion of a Dacron patch in two. The tumour volume of the patients ranged from 4.2 to 63.7 cm^3^ (mean: 20.3 ± 12.7 cm3). The tumour volume of those with cerebral symptoms was significantly higher than in the other patients (median: 23.1 vs 14.3 cm^3^, *p* = 0.015).

Ten patients had developed AF after surgery. The characteristics of the patients are shown in Table 1, while Tables 2 and 3 show their pre-operative electrocardiographic and echocardiographic parameters. The patients who developed AF postoperatively were significantly older than those who did not develop AF (median: 61.5 vs 46 years, *p* = 0.009). Among the electrocardiographic parameters, only Pd differed significantly between AF and non-AF patients (median: 57.6 vs 39.8 ms, *p* = 0.004). The LV ejection fraction (median: 62.5 vs 65%, *p* = 0.019) and mean E/A (median: 0.8 vs 1.3, *p* = 0.05) were lower in the AF group than in non-AF patients. The tumour volume was similar in AF and non-AF patients.

**Table 1:** The clinical characteristics of the patients

	*Postoperative AF group (n = 10)*		*Non-AF group (n = 26)*		*p-value*
	*Median*	*Min–max*	*Median*	*Min–max*	
Age (years)	61.5	42–79	46	20–72	0.009
Body mass index (kg/m2)	28.5	20.7–35.1	25.9	17.9–41.1	0.168
Hypertension, n (%)	6 (60)		6 (23.1)		0.053
Diabetes, n (%)	1 (10)		3 (11.5)		1.00
Hyperlipidaemia, n (%)	1 (10)		1 (3.8)		0.484

AF: atrial fibrillation; Max: maximum; Min: minimum.

**Table 2:** The pre-operative electrocardiographic parameters of the patients

	*Postoperative AF group (n = 10)*		*Non-AF group (n = 26)*		*p-value*
	*Median*	*Min–max*	*Median*	*Min–max*	
Heart rate (beats/min)	76.5	64–127	85.5	53–109	0.349
P-wave amplitude (mV)	1.5	0.93–2.72	2.05	0.81–3.64	0.129
P-wave duration (ms)	124	99.6–129.2	112.4	57.6–134	0.069
P-wave dispersion (ms)	57.6	41.2–71.6	39.8	17.2–70	0.004
QTc dispersion (ms)	50	40–100	40	40–130	0.124
Increased P-wave dispersion (n)	10		9		< 0.001

AF: atrial fibrillation; Max: maximum; Min: minimum; QTc: corrected QT interval.

**Table 3:** The pre-operative echocardiographic parameters of the patients

	*Postoperative AF group (n = 10)*		*Non-AF group (n = 26)*		*p-value*
	*Median*	*Min–max*	*Median*	*Min–max*	
LA diameter (mm)	42	31–51	37	29–60	0.147
LV end-diastolic diameter (mm)	48.5	45–64	48	38–64	0.241
LV end-systolic diameter (mm)	31	23–55	29	23–40	0.107
LV ejection fraction (%)	62.5	30–65	65	50–80	0.019
E/A	0.8	0.67–1.50	1.3	0.6–1.71	0.05
Tumour size (mm3)	21.2	9.4–63.7	17.2	4.2–51.3	0.331

AF: atrial fibrillation; E/A: early/late diastolic peak flow velocity; LA: left atrium; LV: left ventricle; Max: maximum; Min: minimum.

The pre-operative and postoperative ECG findings are listed in Table 4. P-wave amplitude, duration and Pd differed significantly after the surgical procedure (*p* < 0.001, *p* = 0.001 and *p* < 0.001, respectively).

**Table 4:** The electrocardiographic parameters of the patients one day before and one week after surgery

	*Pre-operative*		*Postoperative*		*p-value*
	*Median*	*Min–max*	*Median*	*Min–max*	
Heart rate (beats/min)	82	53–127	86.5	64–144	0.606
P-wave amplitude (mV)	1.98	0.81–3.64	1.28	0.64–2.17	< 0.001
P-wave duration (ms)	117.2	57.6–134	98.4	70.8–126	0.001
P-wave dispersion (ms)	50.5	17.2–71.6	30	10–60	< 0.001
P–R interval (ms)	160	110–240	150	90–230	0.063
QRS interval (ms)	90	80–98	90	70–130	0.837
QTc dispersion (ms)	50	10–130	40	10–90	0.437

Max: maximum; Min: minimum; QTc: corrected QT interval.

We modelled a logistic regression analysis to determine the independent predictors of postoperative AF. Age, LA dimension, tumour volume, aortic cross-clamping time and Pd were included in the model. Logistic regression analysis revealed Pd (OR: 1.11, 95% CI: 1.003–1.224, *p* = 0.043) and age (OR: 1.13, 95% CI: 1.001–1.278, *p* = 0.048) as independent predictors of postoperative AF in our cohort of patients.

## Discussion

This study indicated that postoperative AF may also occur after the excision of the tumour in patients with LA myxoma. LA myxomas may cause severe mitral valve stenosis.^22^ Atrial arrhythmias such as AF or flutter may also be identified in patients with atrial myxoma.^23^ Large myxomas may almost fully occupy the atrial outflow and lead to increased LA pressure.^24^ As a result, obstructing atrial outflow and atrial arrhythmias could contribute to elevated LA pressure and dilated LA cavity.

Atrial overload or ventricular hypertrophy, which secondarily increased the chamber diameter and altered conduction, could lead to abnormal electrocardiography findings.^25^ Also tumour size may have been responsible for the changes on ECG.^26^ Harikrishnan *et al.*^21^ showed that larger tumour size correlated with LA enlargement on ECG in patients with LA myxoma. They also showed that evidence of LA enlargement on ECG disappeared in most patients after excision of the tumour. However, Aggarwal *et al.*^27^ found no correlation between tumour size and signs of LA enlargement on ECG. They found that only 35% of the patients with myxoma had signs of LA enlargement on ECG.

In our study, we found that neither tumour volume nor LA dimensions correlated with postoperative AF. However, pre-operative Pd and age were independent predictors of postoperative AF in our cohort. We also found that P-wave duration, amplitude and Pd were significantly shortened after tumour resection.

Abnormal P-wave morphology reflects abnormality of LA size and LA structural abnormalities.^13^ Previous reports^8^,^28^ showed that age and LA dimension are independent predictors for occurrence of AF after cardiac surgery. However, a prior study has demonstrated that age and LA dimension were not as powerful as abnormal P-wave morphology.^13^

Similar to previous studies,^8^,^13^,^28^ our results suggested that abnormal P-wave morphology was the main independent predictor for the development of postoperative AF but the aetiology of AF following cardiac surgery was multifactorial. Pre-operative factors such as age, previous rheumatic fever, hypertension, coronary syndromes, LV hypertrophy, LA enlargement, history of congestive heart failure, electrolytic imbalance, obesity, male gender, chronic obstructive pulmonary disease,^29^ and surgical factors such as traumatic laceration of the atrial tissue (suture line, haematoma and other traumatic causes)^30^ may increase the incidence of postoperative AF.

LV diastolic dysfunction led to an increase in LV end-diastolic diameter and LA pressure. The elevated atrial pressure dilates the atrium and triggers non-homogeneous fibrosis, which changes the shape and geometry of the atrium. All these changes may induce atrial arrhythmias, especially atrial fibrillation.^31^,^32^ P-wave dispersion was also demonstrated to be influenced by elevated LA pressure.^33^

In our study, LV diastolic function was impaired in patients with postoperative AF. Although statistically non-significant, tumour volumes of postoperative AF patients were higher, suggesting a positive effect on atrial pressure. Higher atrial pressure may prolong the duration and dispersion of the P wave in this patient group. There was no difference between patient groups in terms of LA dimensions, which may have been a result of inaccurate measurement. LA volume or multiplane dimension measurements could clarify our results.

Maximal P-wave duration and Pd have been shown to be a non-invasive predictor of AF in patients with mitral and aortic stenosis, dilated cardiomyopathy, acute myocardial infarction, and atherosclerotic heart disease.^34^,^35^ However, there has been no study evaluating the predictive value of Pd for postoperative AF in patients with LA myxoma. Our study suggests a significant association between postoperative AF and pre-operative Pd values in these patients. All patients who developed AF postoperatively had significantly increased Pd (more than 40 ms).

We also found that patients who developed postoperative AF were significantly older than non-AF patients. Previous reports estimated a 24% increase in the incidence of new-onset postoperative AF with each additional five years of age.^36^ Age-related degenerative change and electrophysiological abnormality of atrial cells are the main causes of post-CABG AF in advanced age, mainly patients older than 70 years of age.^37^,^38^

Cardiac myxomas are the most common primary tumour of the heart, and roughly 90% of the tumours are located in the atria, with the LA accounting for 80% of those.^25^ The most common symptom is dyspnoea, followed by palpitation.^39^ Atrioventricular valve and outflow tract obstruction, and AF may contribute to dyspnoea and palpitation. Dyspnoea was the most common reported symptom in our study.

Symptoms depend on the size, form, mobility and location of the tumour.^40^ The obstruction, mainly caused by large, pedunculated tumours, can decrease cerebral flow and lead to syncope. Also the risk of embolism is higher for polypoid or multilobular tumours.^41^ Twelve patients presented with cerebral symptoms in our study and their tumours were larger than those without cerebral symptoms.

## Study limitations

The retrospective design of our study and the small sample size were limitations. Third, there was no long-term Holter monitoring for the detection of AF episodes. Continuousrhythm Holter monitoring during the intensive care period, and telemetry monitoring up to discharge may be a more accurate method to detect transient episodes of AF during hospital stay. Fourth, tumour volume was calculated with the assumption that the tumour was spherical in shape.

## Conclusion

This study showed a high incidence of postoperative AF following surgery in patients with LA myxoma. To identify patients at risk for AF after surgery, Pd is an independent predictor and can be used for patient risk stratification.
